# The Fabrication of Polyimide-Based Tunable Charge Traps Ternary Memristors Doped with Ni-Co Coated Carbon Composite Nanofibers

**DOI:** 10.3390/polym16212993

**Published:** 2024-10-25

**Authors:** Yuanyuan Liu, Liyuan Liu, He Zhao, Jinghua Yin

**Affiliations:** 1 School of Civil and Construction Engineering, Harbin University, Harbin 150086, China; liuyuanyuan@hrbu.edu.cn; 2 Key Laboratory of Engineering Dielectric and Its Application Ministry of Education, Harbin University of Science and Technology, Harbin 150080, China; hezhao@hrbust.edu.cn; 3 Department of Intelligence and Electrical Electronic Engineering, Harbin Cambridge University, Harbin 150069, China

**Keywords:** polyimide composites, multilevel switching behavior, interface layer, nickel-cobalt coated carbon composite nanofibers

## Abstract

In the dynamic fields of information science and electronic technology, there is a notable trend towards leveraging carbon materials, favored for their ease of synthesis, biocompatibility, and abundance. This trend is particularly evident in the development of memristors, benefiting from the unique electronic properties of carbon to enhance device performance. This study utilizes sensitized chemical evaporation and spin-coating carbonization techniques to fabricate nickel-cobalt coated carbon composite nanofibers (SC-NCMNTs). Novel polyimide (PI) matrix composite memory devices were fabricated using in situ polymerization technology. Transmission electron microscopy (TEM) and micro-Raman spectroscopy analyses validated the presence of dual interface structures located between the Ni-Co-MWNTs, carbon composite nanofibers, and PI matrix, revealing a significant number of defects within the SC-NCMNTs/PI composite films. Consequently, this results in a tunable charge trap-based ternary resistive switching behavior of the composite memory devices, exhibiting a high ON/OFF current ratio of 10^4^ and a retention time of 2500 s at an operating voltage of less than 3 V. The mechanism of resistive switching is thoroughly elucidated through a comprehensive charge transport model, incorporating molecular orbital energy levels. This study provides valuable insights for the rational design and fabrication of efficient memristors characterized by multilevel resistive switching states.

## 1. Introduction

Under the drive of high-density information storage requirements, extensive attention has been paid to resistive switching random-access memory (RRAM) based on a metal–insulator–metal (MIM) sandwich structure, attributing to it the advantages of easy processability [1], high flexibility [2,3], 3D stacking, low operating power consumption, and low cost [4,5,6,7]. Since memory units are the most essential building blocks for electronic devices, various types of next-generation memory devices have been developed to overcome physical limitations and low storage capacity [8,9]. Functional polymer materials have shown great application potential in the field of information storage. Nevertheless, the majority of polymer memory devices are restricted to binary storage and exhibit limited capacity [10]. Compared with inorganic semiconductor memory devices, which are rarely reported in the literature due to the difficulty of reliably controlling multilevel storage, organic polymer composite materials have broad application prospects in organic memory [11,12,13]. For example, polymer composite resistive memory devices embedded with carbon composite materials overcome the above-mentioned limits, primarily including metal nanoparticles [14,15], PCBM [16], graphene [17], fullerene [18], and carbon nanotubes (CNTs) [19,20,21]. In addition, the development of multilevel polymer composite memristors offers a significant potential to attain storage capacities surpassing 2 bits per cell [22], thereby facilitating the achievement of high-density data storage. For instance, Liu et al. first fabricated a polymer composite multilevel memory device doped with carbon composite nanofibers (CCNFs) by using electro-spinning and carbonization techniques [23,24]. Alternatively, carbon composite nanofibers, due to their unique characteristics such as large surface area [25], high mechanical strength [26], excellent electrical conductivity [27], and superior flexibility [28], are well-suited for nanoscale fabrication techniques. These properties facilitate the precise and reproducible integration of memristive device arrays into complex nanoelectronic systems, making them highly suitable for future high-density memristor applications [29,30]. Furthermore, enhancing compatibility to facilitate efficient charge transport within memristors can lead to reduced power consumption in polymer matrix memory devices [31]. Despite the notable advantages and potential future applications of multilevel polymer composite memristors, their fabrication has been limited by a lack of appropriate materials. Furthermore, there is a notable paucity of comprehensive studies on the charge-trap mechanism within polymer matrices doped with one-dimensional metal-coated carbon composite nanofibers.

By using the sensitized chemical evaporation and in situ polymerization method, a novel type of nickel-cobalt coated carbon composite nanofiber was synthesized to address the limitations of the resulting material. Using the composite films as active layers to fabricate the memristors with a sandwich structure of ITO/SC-NCMNTs/PI/Al demonstrates excellently tunable multilevel resistive switching behaviors. Physical models of tunable charge traps are proposed to investigate the influence of doping metal-coated SC-NCMNTs microstructures on multilevel switching behaviors, with a focus on analyzing the charge traps through the distribution of traps in diverse microstructures.

## 2. Materials and Methods

### 2.1. Materials

The MWNTs (with average outer and inner diameters of 5–15 nm and 2~5 nm, purity of >98%, and length of 10–30 um) were provided by Chengdu Organic Chemicals Co., Ltd. (Chengdu, China). N, N-dimethylacetamide (DMAC), 4,4′-oxy dianiline (ODA), and Pyromellitic dianhydride (PMDA) were purchased from Sigma Aldrich (St. Louis, MO, USA). The analytical purity of nickel sulfate (NiSO_4_·6H_2_O) was provided by Beijing Beihua Fine Chemicals Co., Ltd. (Beijing, China). The analytical purity of sodium hypophosphite (NH_2_PO_2_H_2_O), ammonium sulfate [(NH_4_)_2_SO_4_], concentrated sulfuric acid (purity of >98%), concentrated nitric acid (purity of purity of >65%), and silver nitrate (AgNO_3_) were purchased from Tianjin Damao chemical reagent Co., Ltd. (Tianjin, China). All the other solvents and experimental materials were provided by Sinopharm Chemical Reagent Co., Ltd. (Beijing, China).

### 2.2. Preparation of Nickel-Cobalt Coated Multi-Walled Carbon Nanotubes

Through a sensitized and activated electroless plating process, the nano Ni-Co coated carbon nanotubes (Ni-Co-MWNTs) were prepared. The fabrication procedure is as follows: (a) The carbon nanotubes were calcined at 520 °C for 2 h, and then mixed into a mixture flask with concentrated nitric acid and concentrated sulfuric acid (1:3 volume ratio). (b) The mechanical stirrer in an 80 °C water bath and ultrasonic waves were simultaneously applied for 2 h until we obtained stable suspensions. The resulting mixture was diluted with distilled water and set aside to obtain a neutral mixture by repeatedly washing with a large amount of distilled water. (c) After drying at 80 °C and grinding, the nano-sensitized carbon nanotubes were obtained. (d) Silver nitrate solid powder weighing 33.9 g was dissolved in 50 mL distilled water to produce a saturation solution at room temperature. Ammonia water was then added to this solution until turbidity was observed, followed by the addition of more ammonia water until the solution became clear again. The addition of ammonia water was ceased at this point to obtain a silver-ammonia complex solution. (e) A certain amount of carbon nanotubes were dissolved and sensitized in the resulting silver-ammonia solution by using an ultrasonic wave for 1 h. Subsequently, the mixture was filtered and washed with distilled water to neutralize. Thereafter, the sensitized carbon nanotube samples were prepared by drying at a constant temperature in a drying chamber, followed by grinding. (f) Based on the basic formula of electroless plating, we mixed the solution of sodium citrate (Na_3_C_6_H_5_O_7_·H_2_O, purity >80 g/L) and ammonium sulfate (NH_4_SO_4_, purity >65 g/L) into the solution of nickel sulfate and cobalt sulfate. After uniformly mixing, the sensitized carbon nanotube samples were added into the mixture solution to disperse with ultrasonic waves for 30 min. (g) We then added the prepared sodium hypophosphite solution to continue dispersing for 10 min before adjusting the bath pH at a certain temperature of water bath. When there were no more bubbles appearing in the solution, the reaction stopped. After cooling the solution, the samples were washed to neutralize with a magnet and dried at constant 80 °C. (h) The Ni-Co-MWNTs samples after plating were obtained by keeping them at 500 °C for 2 h. The contents of the Ni and Co metals in the synthesized Ni-Co-MWNTs samples were quantified as 0.68 wt% and 0.14 wt%, respectively. These measurements were conducted using the American Thermo Scientific iCAP RQ (MS)/iCAP PRO (AES), hereafter referred to as ICP-AES/MS, under the testing conditions detailed in the Supporting Information (SI)

### 2.3. Preparation of Electro-Spinning Carbonization Samples

**Figure 1:**
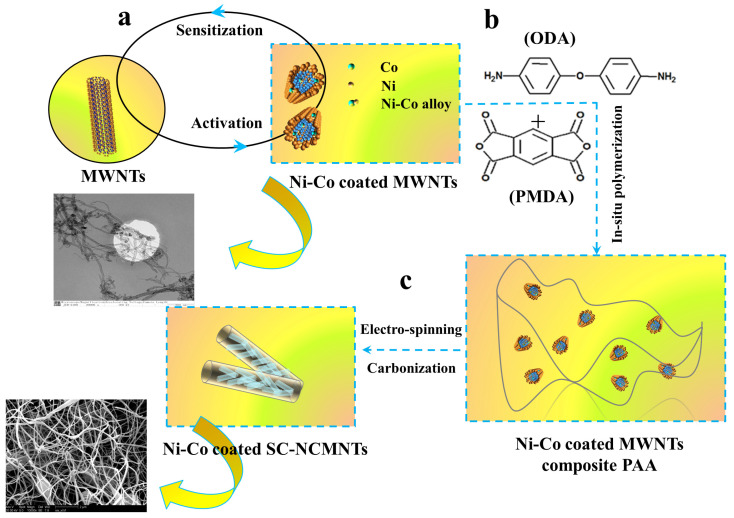
Preparation flow charts and SEM image of (**a**) Ni-Co coated MWNTs. (**b**) Ni-Co coated MWNTs composite PAA. (**c**) Ni-Co coated SC-NCMNTs.

Using an in situ polymerization method and spin coating carbonization technique, nickel-cobalt-coated carbon composite nanofibers were prepared by doping the resulting Ni-Co-MWNTs, as shown in Figure 1. The synthesis procedure is as follows: (a) 0.03156 g Ni-Co-MWNTs and DMAC were added into a three-opening flask with a stirrer, and then the flask was placed in an ultra-sonic bath. The mechanical stirrer and ultrasonic waves were simultaneously applied until we obtained stable suspensions. (b) ODA of 3.6227 g was added into the flask to dissolve in the suspensions by applying mechanical stirring and ultrasonic waves for 2 h. (c) PMDA of 4.0673 g was divided into five portions, which were then added into the suspension and continuously stirred for 4 h until mixed sufficiently to obtain polyamic acid (PAA). (d) We injected the PAA mixture into a 10 mL syringe and fixing the syringe on a booster. (e) We performed high-voltage electro-spinning using a QZNT-E04 electro-spinning machine in the voltage conditions of 15.0–17.0 kV and the liquid supply rate of 0.72 mL/s. The rotation speed of the roller and reciprocating speed were 150 r/min and 50 cm/min, respectively, the temperature was 20 °C, and the humidity was 33% RH. (f) The obtained spinning sample was coped with gradient heating according to Table 1 and imidization treatment using a 350 °C blast oven. (g) Thereafter, using a clean ceramic crucible, we located the imidized sample in the center of the tube furnace. (h) Under an argon gas protection from oxygen, carbonization treatment is carried out by gradient heating of 8 °C/min up to 800 °C, and then kept at 800 °C for 8 h. (i) The carbonization sample was gradient cooled to room temperature over the course of 2 h. Finally, the samples of spinning-carbonization Ni-Co-MWNTs/polyimide (PI) and PI were obtained, named as SC-NCMNTs and SC-PI, respectively. The composition of spinning-carbonization Ni-Co-MNTs/PI samples was analyzed using ICP-AES/MS. The ICP-AES/MS analysis, with testing conditions outlined in the SI, quantified the contents of Ni, Co, and MWNTs in the synthesized samples, as presented in Table 2.

### 2.4. Preparation of Storage Devices

The SC-NCMNTs/PI was synthesized through the different content doping of carbon composite nanofibers using an in situ polymerization process. The composite solution was centrifuged for 5 min at 3000 revolutions per minute to separate the upper clear liquid. The supernatant was then evenly applied onto indium tin oxide (ITO) glass using a spin coating machine at a speed of 7000 revolutions per minute, followed by curing at 80 °C for 8.0 h. Subsequently, the sample underwent a gradual heating process to achieve imidization from polyacrylic acid PAA to polyimide, with the specific heating steps outlined in Table 1. The prepared film thickness was about 1.2 μm. Subsequently, a circular aluminum electrode with a diameter of 100 μm was vapor-deposited on the sample surfaces by using a vapor deposition process to fabricate storage devices with different doping SC-NCMNTs contents of 0.25 wt%, 0.5 wt%, 1.0 wt%, and 2.0 wt%, with designated memory devices SC-NCMNTs/PI-0.25, SC-NCMNTs/PI-0.5, SC-NCMNTs/PI-1.0, and SC-NCMNTs/PI-2.0.

### 2.5. Characterization Techniques

To study the phase properties of the composite films, the synthesized samples were examined using X-ray diffractometry (XRD, D/max-rB X-ray diffractometer). The morphologies of the resulting samples were analyzed by Scanning Electron Microscopy (SEM), while their microscopic structure was examined with Transmission Electron Microscopy (TEM). Fourier Transform Infrared Spectroscopy (FT-IR) was utilized for a quantitative analysis of the molecular structure of the samples at a resolution of 4 cm^−1^. The optical band gap of the composite films was determined by measuring the UV-vis diffuse reflectance spectra (UV-vis DRS) using a Perkin-Elmer Lambda-35 UV-vis spectrometer. The resistive characteristics of the printed devices were evaluated based on ITO/SC-NCMNTs/PI/Al sandwich structure. This evaluation was conducted at room temperature without encapsulation using a Keithley 4200 SCS semiconductor parameter analyzer with a probe station.

## 3. Results and Discussion

### 3.1. Electrical Characterization

**Figure 2:**
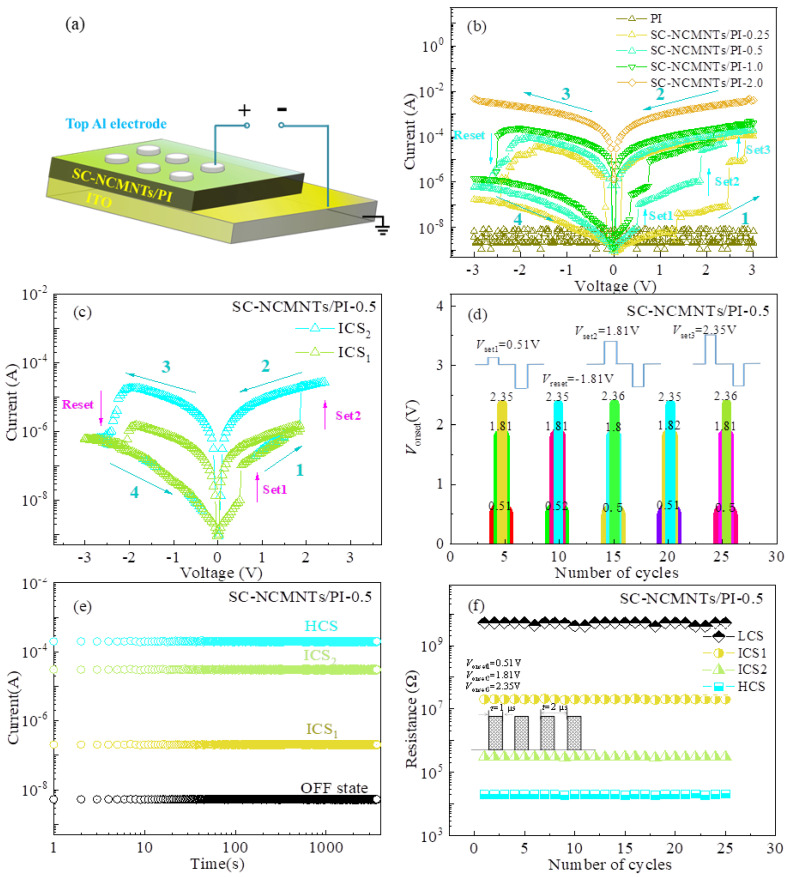
(**a**) Device model. (**b**) I-V curves. (**c**) I-V curves under the ICS_1_ and ICS_2_. (**d**) The threshold voltage reproducibility. (**e**) The limited test of retention time. (**f**) Endurance of the SC-NCMNTs/PI-0.5 memory device.

The I-V characteristics of the device demonstrate a ternary resistive switching behavior, wherein the current gradient escalates with an increase in positive bias, suggesting the presence of multilevel resistive switching behavior. To examine their electrical properties, the devices were operated under a cycle voltage scan of −3 V→0 V→+3 V→0 V→−3 V, as shown in Figure 2a.

As shown in Figure 2b, the SC-NCMNTs/PI devices with low SC-MWNTs doping (≤2.0 wt%) displayed clear multilevel resistive switching behaviors at room temperature. Three abrupt current jumps can be observed for the SC-NCMNTs/PI devices maintaining four conductance states, including a high conductance state (HCS, indicative of low resistance), two intermediate conductance states (ICSs) and a low conductance state (LCS, indicative of high resistance), respectively. At a low voltage, the SC-NCMNTs/PI-0.5 device stays at a LCS until the applied voltage reaches thresholds of *V*_onset1_ = 0.5 V, *V*_onset2_ = 1.85 V, and *V*_onset3_ = 2.36 V, at which point their current jumps from an LCS to two ICSs (i.e., ICS_1_ and ICS_2_) and then up to an HCS, referred to as the information writing process [32]. Figure 2b clearly demonstrates that *V*_onset_ decreases as the doping content increases. Conversely, the ON/OFF current ratio (*I*_ON_/*I*_OFF_), which ranges from 10^3^ to 10^4^, exhibits an inverse relationship with the doping contents; specifically, the ratio tends to decrease with higher doping levels. When exerting biased voltages of −3 V→0 V→+1.85 V→0 V→−3 V and −3 V→0 V→+2.36 V→0 V→−3 V, the SC-NCMNTs/PI-0.5 device can remain the ICS_1_ at *V*_onset1_ = 0.5 V and ICS_2_ at *V*_onset2_ = 1.85 V very well without a significant change in threshold voltage in 25 cycles at room temperature, as depicted in Figure 2c,d. In addition, under the same voltage stresses of *V*_onset1_ and *V*_onset2_ and the ON/OFF current state of the devices of SC-NCMNTs/PI-0.25, −0.5 and −1.0 can be sustained up to more than 2500 s, and their endurance under continuous read pulses (pulse period = 2 μs and pulse width = 1 μs) is stable over more than 25 cycles, as shown in Figure 2e,f. As explained above, it is possible to realize a lower consumption and a high-density storage capacity compared with reported multilevel memory devices of carbon nanotube composites at present. When doping 2.0 wt%, the device turns on at very low bias and exhibits no switching behavior within the voltage range is measured.

### 3.2. Microstructure Characterization

**Figure 3:**
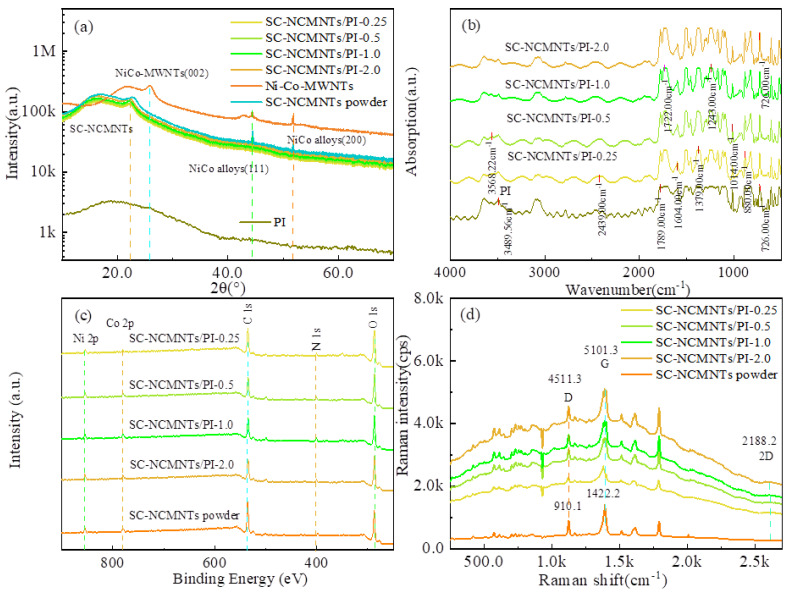
(**a**) XRD. (**b**) FT-IR. (**c**) XPS. (**d**) Raman shift of the PI and SC-NCMNTs/PI composite films.

To analyze the impact of SC-NCMNTs on the microstructure of the composite films and realize the multilevel resistive switching behavior mechanism of the memory devices, the tests of phase characteristics and molecular valence bond structure for the composite films were implemented, as shown in Figure 3. In view of the XRD patterns of Ni-Co-MWNTs, SC-NCMNTs powders, PI, and their composite films, as shown in Figure 3a, the typical peaks of PI in SC-MWNTs/PI composite films exist between 2θ = 18~20° and shift slightly to the left relative to pure PI, with the Ni-Co-MWNTs characteristic peak at 2θ = 22.84°. The Ni-Co-MWNTs characteristic peak can be observed at 2θ = 26.2°, where the characteristic diffraction peaks at the positions of 2θ = 44.44°, 51.77° are consistent with the lattice constants of Ni-Co alloy, corresponding to the face-centered cubic phase (111) and (200), respectively [33,34]. As the concentration of SC-NC-MNTs increased, the intensity of the PI diffraction peaks also increased. However, these peaks remained lower than those characteristic of pure PI. This observation suggests that the incorporation of SC-NC-MNTs leads to a disruption in the ordered arrangement of PI molecular chains and a decline in their orientation, ultimately resulting in a reduction in the crystallinity of the PI matrix. As can be seen from Figure 3a, the composite films doped SC-NCMNTs contents with 0.25 wt%, 0.5 wt%, 1.0 wt%, and 2.0 wt% exhibit remarkable diffraction peaks of SC-NCMNTs at 2θ = 22.04°, 22.06°, 22.12°, and 22.16°, indicating that SC-NCMNTs are incorporated into SC-MWNTs/PI composite films.

Figure 3b shows the FT-IR spectra of the composite films. The FT-IR analysis reveals the presence of symmetric C=O stretching vibrations, C-N stretching vibrations, imide ring C=C stretching vibrations and asymmetric C-O bond stretching vibrations, corresponding to wavenumbers of 726 cm^−1^, 1379 cm^−1^, 1604 cm^−1^, and 1789 cm^−1^, respectively. These findings indicate that the composite films possess a benzene ring conjugated structure, indicating no change in PI characteristics by the doping of the SC-NCMNTs. In addition, the IR absorption intensity of the symmetrical vibrations of C=O and C=C of imide rings in the SC-NCMNTs/PI composite films is obviously strengthened with increasing content in the wavelength range of 1300 cm^−1^~1700 cm^−1^. The acylamide groups (-NH_2_-) were produced through a sensitization process involving a silver-ammonia complex solution. This process resulted in a medium-intensity absorption peak and a strong absorption peak at 2439 cm^−1^ and 1604 cm^−1^, respectively. The -NH_2_- group, possessing charged lone electron pairs, can interact with the carboxyl group on the PI molecular chains within the composite films [35], thereby influencing charge transmission [36].

As shown in Figure 3c, the XPS spectrum peaks of Ni and Co coexist in a metal state corresponding to 854.97 eV and 780.67 eV, respectively. Additionally, there are a set of peaks corresponding to C 1s, N 1s, and O 1s in the XPS scan spectra, where the N 1s signal indicates the presence of pyridinic-N (398.2 eV) and pyrrolic-N (400.1 eV), as displayed in the zoomed-in N-XPS of composite films in Figure S1 [24], which is consistent with the results for other N-doped carbon materials [37,38]. The bonding of Pyridinic-N with two C atoms and one localized p-electron in the π conjugated system, along with the bonding of Pyrrolic-N with two p-electrons [39], indicates that doping SC-NCMNTs with N atoms introduces charged lone electron pairs into the PI, leading to the formation of -NH_2_- between the SC-NCMNTs with N atoms and the PI molecular chains [40].

To analyze the impact of Ni-Co-MWNTs on the microstructure of the composite films, the micro-Raman spectrum tests of Ni-Co-MWNTs, SC-NCMNTs, and SC-NCMNTs/PI were given in Figure 3d, displaying the characteristic D and G bands of Ni-Co-MWNTs. The *I*_D_/*I*_G_ intensity ratio, commonly utilized as an indicator of defect density in Ni-Co-MWNTs and its composite films, was observed to be 0.64 and 0.88 in the cases of SC-NCMNTs powder and SC-NCMNTs/PI-0.5 film, respectively. This suggests the presence of notable structural defects of atoms within Ni-Co-MWNTs. Furthermore, the doping of SC-NCMNTs in the composite films resulted in the introduction of a significant number of defects in the materials [41].

**Figure 4:**
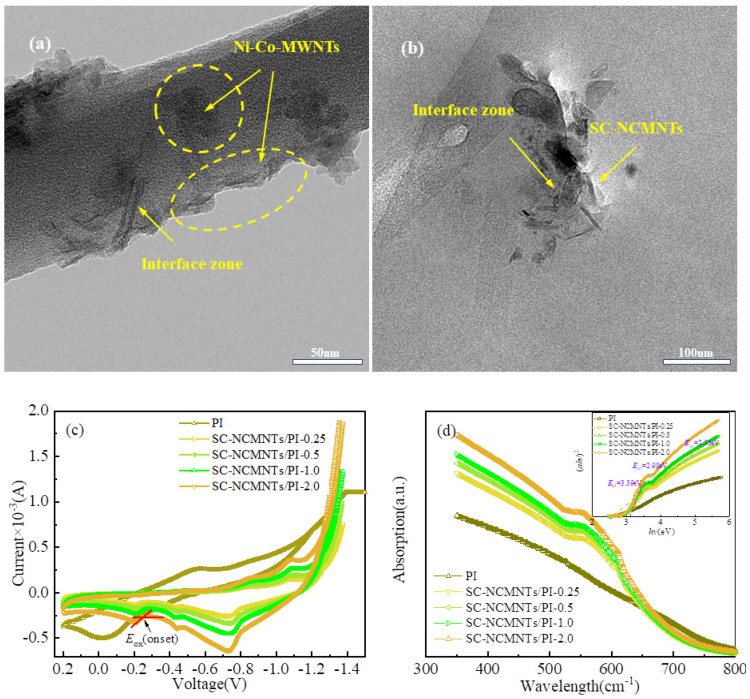
TEM images of (**a**) SC-NCMNTs. (**b**) SC-NCMNTs/PI. (**c**) Cyclic voltammetry. (**d**) UV-vis DRS and Kubelka-Munk plots of the PI and SC-NCMNTs/PI composite films.

To observe the microstructures of composite films and SC-NCMNTs, the TEMs of SC-NCMNTs and corresponding composite films are shown in Figure 4a,b, where the diameters of SC-NCMNTs are in the range of 110–130 nm. As depicted in Figure 4a, the distribution of Ni-Co-MWNTs both internally and externally on the surfaces of SC-NCMNTs results in the formation of a transition zone with a thickness significantly exceeding that of the MWNTs. This observation suggests the presence of an interface zone (IZ) between the Ni-Co-MWNTs and SC-NCMNTs. Additionally, Figure 4b illustrates the dispersion of SC-NCMNTs within the PI matrix, revealing the formation of numerous fuzzy IZs measuring about 5.0 nm between the SC-NCMNTs and the PI matrix. Thus, the doping of SC-NCMNTs results in double IZs in SC-NCMNTs/PI composite films. The formation of IZs reinforces the effect of the carrier transport on the resistive switching behavior of memristors.

The energy levels of molecular orbitals are closely related to the conductance behavior of memory devices. Furthermore, the molecular orbital energy level (MOEL) is significantly influenced by the distribution of IZs and defects acting as charge traps. The cyclic voltammeter (testing conditions are detailed in the SI) and the UV-vis DRS of the PI and SC-NCMNTs/PI composite films were analyzed, as depicted in Figure 4c,d. The initial oxidation peak *E*_OX(onset)_ and the optical band gap (*E*_g_) were determined, revealing three *E*_g_ values for the SC-NCMNTs/PI composites. From the UV-vis DRS and cyclic voltammeter tests, the MOEL of the SC-NCMNTs/PI composite films can be calculated according to the theoretical equations given as [42,43]:

*E*_HOMO_ = −[*E*_OX_(onset) − *E*_reference_ + 4.80] (1)

*αhv* = B(*hv* − *E*_g_)^*n*^ (2)

*E*_LUMO_ = *E*_HOMO_ + *E*_g_ (3)

where *E*_HOMO_ is the highest occupied MOEL, *E*_LUMO_ is the lowest unoccupied MOEL, *α* represents the absorption coefficient, *h* denotes Planck’s constant, indirect transition coefficient *n* = 2, and B is a constant. All of these variables for the composite films can be calculated using Hg/HgSO_4_ (the reference potential *E*_reference_ = 0.24 eV) as a reference electrode.

As the doping content increases, the highest occupied molecular, HOMO_1_, increases and HOMO_2_ decreases slightly. Concurrently, the lowest unoccupied molecular orbital energy levels (LUMO) increase in a manner consistent with Equation (3). Specifically, LUMO_1_ is associated with charge transport in the interface zone (IZ_1_), which is situated between the SC-NCMNTs and the PI matrix. In contrast, LUMO_2_ and LUMO_3_ are linked to surface-state defects of the Ni-Co-MWNTs in the interface zone (IZ_2_), located between the Ni-Co-MWNTs and the SC-NCMNT, and internal metal atom defects (IDs) within the Ni-Co-MWNTs, respectively.

### 3.3. Resistive Mechanism of the Composite Memory Devices

**Figure 5:**
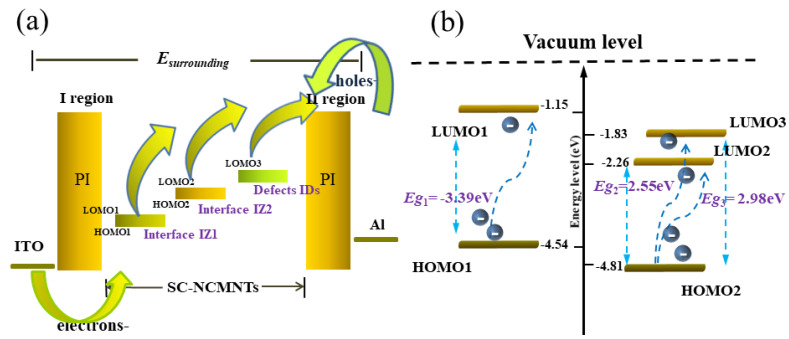
(**a**) The charge transport model incorporating with MOELs at *V*+_onset1_. (**b**) The single-double type MOELs for SC-NCMNTs/PI-0.5 memory device.

Considering that both IZs and defects influence carrier transport in composite films, it is essential to examine the impact of IZs on the resistive properties of polyimide matrix functional layer materials doped with SC-NCMNTs. Furthermore, the formation of Ni-Co-MWNTs inevitably introduces internal defects between the MWNTs and Ni-Co alloy. To elucidate the resistive switching mechanism of composite memristors, the carrier transport within IZs and internal defects IDs is analyzed to provide a plausible explanation for the observed multilevel switching behavior. The TEM test and Raman spectrum analysis of the IZs and IDs within the composite films present compelling evidence for charge transport molecular orbital models. These models encompass a complex charge transport model incorporating with MOELs at *V*+_onset1_ characterized by a sandwiched structure and a single-double type MOEL of SC-NCMNTs/PI-0.5 memory device, as depicted in Figure 5a,b. The composite memristor can be theoretically conceptualized as an assembly of micro-devices (AMDs) connected in parallel, exhibiting several analogous model regions of SC-NCMNTs. Figure 5a illustrates the SC-NCMNTs/PI composite, highlighting two distinct IZs, identified as IZ_1_ (situated between the SC-NCMNTs and the PI matrix) and IZ_2_ (located between the Ni-Co-MWNTs and the SC-NCMNTs). The IDs represent the internal metal atom defects within the Ni-Co-MWNTs. These observations are corroborated by the TEM images presented in Figure 4a,b, as well as the Raman shift shown in Figure 3d.

From the results of the FI-IR test, a substantial number of defects were formed in IZ_1_, characterized by numerous shallow trap centers. The surface state defects of the Ni-Co-MWNTs, along with their internal metal atom defects, introduced deep trap centers (denoted as LUMO_2_ and LUMO_3_) in IZ_2_ and IDs within the Ni-Co-MWNTs. Consequently, the charge transport in IZ_1_, IZ_2_, and IDs is affected by three distinct types of traps, corresponding to LUMO_1_, LUMO_2_, and LUMO_3_, respectively [23]. The charge transports in the IZs and IDs are influenced by three types of traps, leading to varying switching behaviors in memristors. As previously discussed, when an external electric field (*E*_surrounding_) is applied in the sequential order of *V*−→0 V→*V*+→0 V→*V*− to the top Al electrode of the memristor, an AMD in the functional layer is circulated by the surrounding *E*_surrounding_. As shown in Figure 5a, regions I and II represent PI substrates, while the central region corresponds to Ni-Co-MWNTs with the IZs and IDs. Based on the FT-IR test results, significant quantities of NH_3_ were generated in IZ_1_. The surface state defects of Ni-Co-MWNTs and the internal metal atoms contribute to the formation of deep trap centers in IZ_2_ and within the Ni-Co-MWNTs. During the initial voltage sweep from *V*−→0 V, since the energy level difference *φ*_A_ between ITO and PI is less than the *φ*_B_ between Al electrode and PI matrix [44,45,46], a substantial number of holes are injected from the AMDs closed to ITO into the highest occupied molecular orbit energy levels of HOMO_1_ and HOMO_2_ near region I. A limited number of electrons are introduced into the PI matrix and the IZs from the adjacent AMDs near the Al electrode. Initially, the electrons are injected into LUMO_1_ of IZ_1_ and then into LUMO_2_ and LUMO_3_ in the IZ_2_ and IDs of Ni-Co-MWNTs. This process results in the formation of a built-in electric field characterized by a positively charged zone in region I and a negatively charged zone in region II. A significant concentration of holes in region I impedes electron transport, causing the device to remain in a LCS. During the sequential voltage sweep from 0 V to *V*_+onset1_, a substantial number of electrons are injected from the adjacent AMDs near the ITO electrode into region I, where they recombine with the existing holes. Under the influence of the AMDs’ electric field near the Al electrode, a fraction of the electrons in LUMO_1_, LUMO_2_, and LUMO_3_ from IZ_1_, IZ_2_, and IDs are de-trapped into region II, as depicted in Figure 5a.

The charge de-trapping process involving single-double MOELs for the SC-NCMNTs/PI-0.5 memory device is comprehensively illustrated in Figure 5b. In contrast, a small number of holes are injected into region II. Upon reaching *V*_onset1_, a significant number of electrons are de-trapped from the LUMO_1_ and traverse into the hole-blocking layer of region II to the adjacent region of the Al electrode. This process results in an abrupt increase in current, causing the SC-NCMNTs/PI-0.5 device to transition from an LCS to an ICS_1_. As the voltage continues to increase up to *V*_onset2_, a substantial de-trapping of electrons from the LUMO_2_ occurs, leading to a sharp rise in current and transitioning the device to an ICS_2_. Upon increasing the voltage up to *V*_onset3_, a significant de-trapping of electrons from the LUMO_3_ occurs, leading to a sharp increase in current, transitioning to an HCS. Further increases in the doping content of SC-NCMWNTs result in greater thicknesses of IZs, thereby increasing the charge trap densities in IZ_1_, IZ_2_, and IDs [47]. The current value is directly proportional to the charge trap density provided by the Ni-Co coated carbon nanofiber composites [16]. When subjected to an external electric field, a substantial de-trapping of electrons from IZ_1_, IZ_2_, and IDs enhances the ON/OFF current ratio of the memory devices. With the incorporation of doping content, the decreased separation between nanofibers improves the conductivity of the composite films [48], resulting in a reduction in the threshold voltage *V*_onset_ of memory devices. As previously analyzed, the fabricated memory device exhibits multilevel resistive switching behavior at a low threshold voltage, which corresponds to reduced power consumption.

## 4. Conclusions

In summary, nickel-cobalt-coated carbon composite nanofibers doped by Ni-Co-MWNTs were fabricated by sensitized chemical evaporation and an electro-spinning carbonization method. When the resulting carbon composite nanofibers are doped into a PI matrix, composite films with different doping contents are prepared. Additionally, novel memristors featuring a sandwich structure were successfully designed and fabricated using the SC-NCMNTs/PI films as functional material layers. The fabricated memory devices demonstrated ternary resistive switching behaviors characterized by a high *I*_ON_/*I*_OFF_ ratio of 10^4^, low threshold voltages, and good retention time exceeding 2.5 × 10^3^ s. Physical models were employed to investigate the influence of the interfaces and defects on the three-level switching behaviors of the memory devices. The findings reveal that the microstructures, serving as charge transport layers, significantly affect the charge traps within the memristors. This study on the role of doping metal-coated SC-NCMNTs in storage behaviors suggests the potential for realizing high-density memory devices in a controlled manner in the future.

## Figures and Tables

**Table 1:** The heating steps to remove DMAC and promote imidization from PAA to PI.

Heating Steps	1	2	3	4	5	6	7	8
Temperature (°C)	80	120	150	180	210	270	310	350
Time (h)	8.0	0.5	0.5	0.5	0.5	0.5	0.5	1.0

**Table 2:** The composition of spinning-carbonization Ni-Co-MWNTs/PI samples.

Content (mg/Kg)	SC-NCMNTs/PI-0.25	SC-NCMNTs/PI-0.5	SC-NCMNTs/PI-1.0	SC-NCMNTs/PI-2.0
Ni	14.9	29.82	61.56	123.00
Co	6.69	13.37	26.60	53.42
MWNTs	999,581.09	999,162.18	998,324.22	996,648.44

## Data Availability

The data and methodology are available upon request, pertaining to the characterization conducted using ICP-AES/MS and detailed N-XPS analysis, as well as the measurement of C-V characteristics for the composite films. The original contributions presented in the study are included in the Supplementary Materials, further inquiries can be directed to the corresponding author of liuyuan1363@163.com (L.L.).

## Supplementary Materials

The following supporting information can be downloaded at: https://www.mdpi.com/article/10.3390/polym16212993/s1, Figure S1. The zoomed-in N-XPS of the SC-NCMNTs powder and composite films.
