# Supplementary material for: The Fabrication of Polyimide-Based Tunable Charge Traps Ternary Memristors Doped with Ni-Co Coated Carbon Composite Nanofibers
Source: Polymers (Basel). 2024 Oct 25;16(21):2993. doi: 10.3390/polym16212993 (PMC11548601; doi:10.3390/polym16212993)
Supplement: Supplementary file 1 [file polymers-16-02993-s001.zip › polymers-3207393-supplementary.pdf]

Supporting Information

The Fabrication of Polyimide-based Tunable Charge Traps Ternary Memristors Doped with Ni-Co Coated Carbon Composite Nanofibers

1. The composition characterization of Ni-Co-MWNTs

Under the condition of the sample flush time of 10000 us and RF Power of 1350 W, the characterization of the Ni-Co-MWNTs was conducted using the American Thermo Scientifc-iCAP RQ (MS)/iCAP PRO (AES) (refered as ICP-AES/MS) under the conditions of a nebulizer flow of 0.76 L/min and auxiliary gas of 0.75 L/min. The contents of the Ni and Co metals in the synthesized Ni-Co-MWCNTs samples were determined to be 0.68 wt% and 0.14 wt%, respectively.

2. The composition characterization of spinning-carbonization Ni-Co-MWNTs/PI samples

The characterization of spinning-carbonization Ni-Co-MWNTs/PI samples was performed under a sample flush time of 10,000 μs and an RF power of 1350 W, utilizing the American Thermo Scientific iCAP RQ (MS)/iCAP PRO (AES), hereafter referred to as ICP-AES/MS. The analysis employed a scanning number of 100, with combustion-supporting gas, cooling gas, and atomized gas flow rates set at 0.75 L/min, 14 L/min, and 0.76 L/min, respectively. The ICP-AES/MS analysis quantified the content of Ni, Co, and MWNTs in the synthesized samples, as presented in Table 2.

3. The characterization of the cyclic voltammetry

The cyclic voltammetry (CV) properties of PI and SC-NCMNTs/PI composite films were evaluated using a CH 660Å electrochemical analyzer, employing potassium hydroxide with concentration of 0.1 mol/L as the electrolyte. A composite film with dimensions of 1 cm * 3 cm was utilized as the working electrode, while a calomel electrode served as the reference electrode, and a platinum plate electrode was used as the auxiliary electrode. The potential was scanned from 0.2 V to -1.2 V at a scan rate of 10 mV/s.

4. The zoomed-in N-XPS of composite films

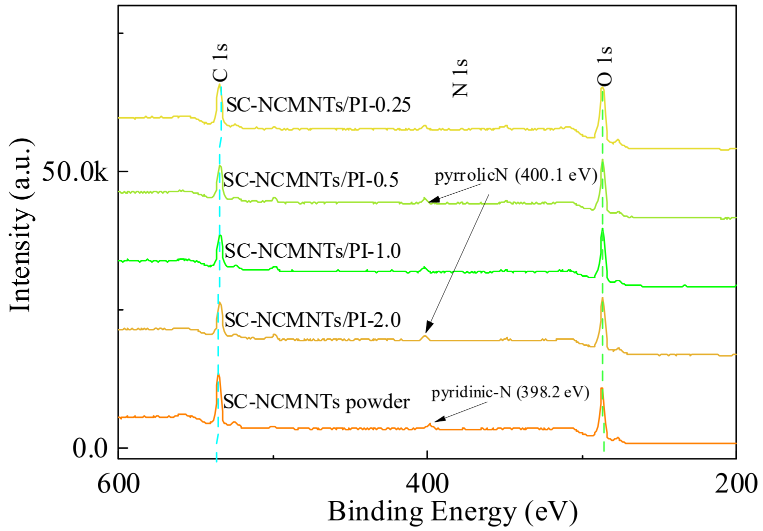

Figure S1. The zoomed-in N-XPS of the SC-NCMNTs powder and composite films.
